# Differentiation of human induced pluripotent stem cells to mature functional Purkinje neurons

**DOI:** 10.1038/srep09232

**Published:** 2015-03-18

**Authors:** Shuyan Wang, Bin Wang, Na Pan, Linlin Fu, Chaodong Wang, Gongru Song, Jing An, Zhongfeng Liu, Wanwan Zhu, Yunqian Guan, Zhi-Qing David Xu, Piu Chan, Zhiguo Chen, Y. Alex Zhang

**Affiliations:** 1 Sanofi-Xuanwu Joint Lab for Regenerative Medicine, Xuanwu Hospital, Capital Medical University, Beijing, 100053, China; 2 Center of Neural Injury and Repair, Beijing Institute for Brain Disorders, Beijing, China; 3 Center of Parkinson's Disease, Beijing Institute for Brain Disorders, Beijing, China; 4 China R&D Center, Sanofi, Beijing 100022, China; 5 Department of Neurobiology, Beijing Key Laboratory of Major Brain Disorders, Beijing Institute of Brain Disorders, Capital Medical University, Beijing, 100069, China; 6 Department of Neurobiology, Xuanwu Hosptial, Capital Medical University, Beijing, 100053, China

## Abstract

It remains a challenge to differentiate human induced pluripotent stem cells (iPSCs) or embryonic stem (ES) cells to Purkinje cells. In this study, we derived iPSCs from human fibroblasts and directed the specification of iPSCs first to Purkinje progenitors, by adding Fgf2 and insulin to the embryoid bodies (EBs) in a time-sensitive manner, which activates the endogenous production of Wnt1 and Fgf8 from EBs that further patterned the cells towards a midbrain-hindbrain-boundary tissue identity. Neph3-positive human Purkinje progenitors were sorted out by using flow cytometry and cultured either alone or with granule cell precursors, in a 2-dimensional or 3-dimensional environment. However, Purkinje progenitors failed to mature further under above conditions. By co-culturing human Purkinje progenitors with rat cerebellar slices, we observed mature Purkinje-like cells with right morphology and marker expression patterns, which yet showed no appropriate membrane properties. Co-culture with human fetal cerebellar slices drove the progenitors to not only morphologically correct but also electrophysiologically functional Purkinje neurons. Neph3-posotive human cells could also survive transplantation into the cerebellum of newborn immunodeficient mice and differentiate to L7- and Calbindin-positive neurons. Obtaining mature human Purkinje cells *in vitro* has significant implications in studying the mechanisms of spinocerebellar ataxias and other cerebellar diseases.

Purkinje cells are the only output neurons in cerebellar cortex and the major target afflicted in spinocerebellar ataxias. Obtaining patient specific Purkinje cells would be a valuable tool to investigate the disease mechanisms. However, although a substantial amount of knowledge has been gained on the regulatory machinery that controls the development of Purkinje cells, it remains a challenge to sufficiently differentiate human embryonic stem (ES) or induced pluripotent stem (iPS) cells to mature Purkinje cells.

To date, most *in vitro* studies on Purkinje cells employed murine cell cultures as a model system. Primary Purkinje cultures can be obtained from embryonic or neonatal mouse and rat cerebellar tissues^1^, and are useful in investigating the cell biology and electrophysiology of Purkinje cells. However, for studies focusing on regenerative medicine and the developmental biology of Purkinje cells, ES and iPS cells have advantages due to the extensively proliferative capacity and the specification process recapitulating the normal differentiation of Purkinje cells. *Fgf8* and *Wnt1* are two key morphogens produced at the isthmic organizer and play essential roles for the genesis and development of cerebellum^2^,^3^,^4^,^5^. Nevertheless, simply adding *Fgf8* and *Wnt* ligand to mouse ES culture only gives rise to a small fraction of Purkinje cells, usually less than 1% of total cells^6^,^7^,^8^.

In 2010, Muguruma et al^9^ reported a new strategy to derive Purkinje cells from mouse ES cells. Instead of adding *Fgf8* and *Wnt* ligand, the authors treated the ES cells with *Fgf2* and insulin in a restricted time window, which can induce a self-sustaining signaling pathway that triggers a high level expression of endogenous *Fgf8* and *Wnt1*. Addition of cyclopamine on differentiation day 7 onward further promotes dorsalization of the cells and the efficiency of Purkinje progenitor cell production.

Despite the progress in mouse work, it remains a challenge to translate it into human work. To date, there has been no publication that reported a successful differentiation from human ES/iPS cells into Purkinje neurons. In the current study, we attempted to achieve this by capitalizing on the information from Muguruma and colleagues' work^9^. Using similar stratigies, we managed to obtain Purkinje progenitor cells from human iPSCs. However, simply co-culturing human Purkinje progenitors with granule cells could not lead to mature functional Purkinje neurons. Only by co-culturing with fetal cerebellar organotypic slices did we succeed to differentiate the progenitor cells into mature Purkinje neurons.

## Results

### iPSC generation and characterization

Primary fibroblast cultures were derived from human skin biopsies and infected with retrovirus encoding the four Yamanaka factors – Oct4, Sox2, Klf4 and c-Myc. Two to 3 weeks post-infection, iPSC colonies emerged and were picked for passaging and characterization. Immunofluorescence staining revealed that the colonies were positive for alkaline phosphatase (AP), Oct4, SSEA-4, TRA-1-81, Nanog, and TRA-1-60 (Fig. S1A–F). RT-PCR analysis showed that the cells had activated the endogenous expression of pluripotent genes *OCT4*, *SOX2*, *KLF4*, *C-MYC*, *NANOG*, *FGF4*, *REX1*, and *DPPA4* (Fig. S1G). Using primers that specifically amplify the exogenous factors, we confirmed genomic incorporation of the Yamanaka factors (Fig. S1H). All the generated iPSC colonies showed a normal karyotype (Fig. S1I) and hypomethylation at the promoters of endogenous *OCT4* and *NANOG* (Fig. S1J and K). To examine whether the induced cells had the capacity to differentiate to the three germ layer cells, we injected the iPSCs into immunodeficient mice and 6–8 weeks later teratoma was observed (Fig. S1L). *In vitro* differentiation of iPSCs through an embryoid body (EB) stage also resulted in cells typically found in ectoderm (Tuj-1+), endoderm (AFP+), and mesoderm (α-SMA+) (Fig. S1L).

### Differentiation of iPSCs to Purkinje progenitors

**Figure 1:**
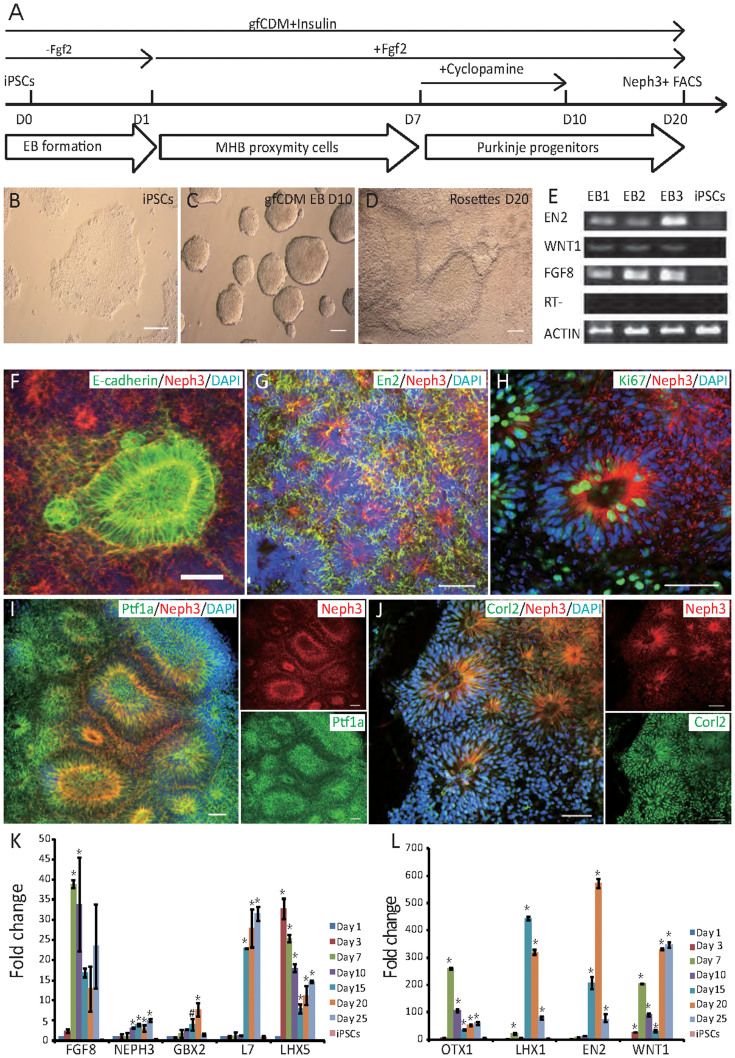
Differentiation of iPSCs to Neph3+ Purkinje progenitors. (A) Schematic representation of the experimental procedure to derive Neph3+ Purkinje progenitors from iPSCs. (B–D) Images of iPSCs (B), floating EBs on Day 10 (C), and neural rosette-like structures on Day 20 (D). Bar = 100 μm. (E) RT-PCR analysis of MHB region-specific gene expression (*FGF8,* *EN2*, and *WNT-1*) in EBs on Day 7. *ACTIN* was amplified as a positive control, and RT (Reverse Transcriptase) minus as a negative control. (F–J) Immunofluorescence staining of neural rosette-like cells on Day 20. Neph3/E-cadherin (F), Neph3/En2 (G), Neph3/Ki67 (H), Neph3/Ptf1a (I), and Neph3/Corl2 (J) were double labeled in some neural rosette-like cells. Bar = 50 μm. (K, L) qPCR analysis of gfCDM EBs and neural rosette-like cells on Days 1, 3, 7, 10, 15, 20, and 25 for MHB specific markers (*EN2*, *GBX2*, *WNT1,* *FGF8* and *OTX1*) and Purkinje cell development associated genes (*NEPH3*, *LHX1,* *LHX5* and *L7*). ANOVA was used for statistical analysis. * p < 0.001 versus Day 1; ^#^ 0.001 < p < Corrected *p*-value 0.007 versus Day 1. All data were analyzed with ANOVA with post Bonferroni test after gene expression was nomalized with HPRT (Fig. 1K) and 18s rRNA (Fig. 1L). The corrected p-value according to Keppel's modified Bonferroni correction was 0.007. The immunostaining experiments were repeated at least three times. The PCR and qPCR were repeated three times as independent tests.

As illustrated in Fig. 1A, iPSCs were cultured on Matrigel (feeder free, Fig. 1B). On Day 0, iPSCs were detached by treatment with Collagenase and re-suspended to form EB-like cell clusters in growth factor-free, chemically defined medium (gfCDM) plus insulin for 24 hrs (Fig. 1C). Insulin was added due to its moderate caudalizing effect^10^. From Day 1 onward, Fgf2 was added to the medium (gfCDM + Insulin), because previous study shows that Fgf2 treatment, in a time sensitive manner, can bias the differentiation towards midbrain-hindbrain regionality^9^. During early cerebellum development, Purkinje cells arise from the alar plate of rhombomere 1. Sonic hedgehog (Shh) that emanates from the floor plate can inhibit Purkinje cell differentiation^9^,^11^. Therefore, cyclopamine, a Shh inhibitor, was added to the culture from Day 7 to Day 10 to promote dorsalization, by passively preventing the cells from ventralization. From Day 10, the aggregates were transferred to adhesive culture dishes to allow attachment and formation of neural rosette-like structures (Fig. 1D). As of mouse cells, addition of Fgf2 on Day 1 can induce endogenous production of midbrain-hindbrain boundary (MHB) organizers - Wnt1 and Fgf8, which can subsequently pattern the cells towards a MHB regionality (En2+)^9^. To address whether the same holds true with human cells, we examined the expression of *EN2*, *WNT1*, and *FGF8*, which are MHB-related genes, on Day 7 of differentiation. In iPSCs, the expression of the above genes was at undetectable levels. However, in cell aggregates treated with insulin and Fgf2 for 7 days, expression of *EN2*, *WNT1*, and *FGF8* was markedly up-regulated (Fig. 1E), suggesting that insulin and Fgf2 treatment may have initiated a self-sustaining signaling pathway that directs the cells to acquire a MHB identity.

During the early development of cerebellar primordium, several markers are expressed by Purkinje progenitors and precursors. Ptf1a is a key transcription factor that is central for the genesis of Purkinje cells and cerebellar GABAergic interneurons. *NEPH3* is a direct target gene of Ptf1a. A majority of Neph3+ cells in cerebellum primordium turn into Purkinje cells later and only a small fraction of Neph3+ cells become Pax2+ GABAergic interneurons^12^. In addition to Ptf1a and Neph3, mitotic Purkinje progenitors also express E-Cadherin and Ki67. Postmitotic Purkinje precursors are positive for Corl2, a transcriptional corepressor that is not expressed in other cerebellar neurons^13^. We examined the expression of the above markers on cultures on Day 20 of differentiation (Fig. 1F–J). The apical (interior) side of the neural rosettes stained positive for E-cadherin, Ptf1a and Neph3, and the basal (exterior) side of the rosettes were positive for Corl2.

We also examined the temporal expression of genes related to Purkinje cell development using real-time PCR (Fig. 1K–L). Cells on Day 1 were used as control since it was the start point of differentiation and most of the genes were at undetectable level in iPSCs at this time point. *LHX5* expression was rapidly brought up on Day 3 and *FGF8* and *OTX1* up on Day 7 compared with those on Day 1 (ANOVA with post Bonferroni test, *p < 0.001). *NEPH3*, *L7*, *GBX2* and *EN2* levels gradually increased over the course of differentiation up to Day 25 or Day 20 (ANOVA with post Bonferroni test, *p < 0.001; 0.001 < ^#^p = 0.003 < corrected p-value 0.007). Expression of *LHX1* exhibited a surge on Day 15 (ANOVA with post Bonferroni test, *p < 0.001). *WNT1* expression increased upon differentiation and showed a fluctuation of expression on Day 10 and Day 15 (ANOVA with post Bonferroni test, *p < 0.001). Despite the fluctuation, even at Day 15, *WNT1* expression was still around 30 fold higher than that at Day 1. All data were analyzed with ANOVA after the gene expression had been normalized with housekeeping genes 18 s rRNA and HPRT. The corrected p-value according to Keppel's modified Bonferroni correction was 0.007. The results confirmed that the differentiation scheme could efficiently drive iPSCs towards MHB regionality and a Purkinje cell lineage.

### Maturation of Purkinje cells by co-culture with organotypic slices

**Figure 2:**
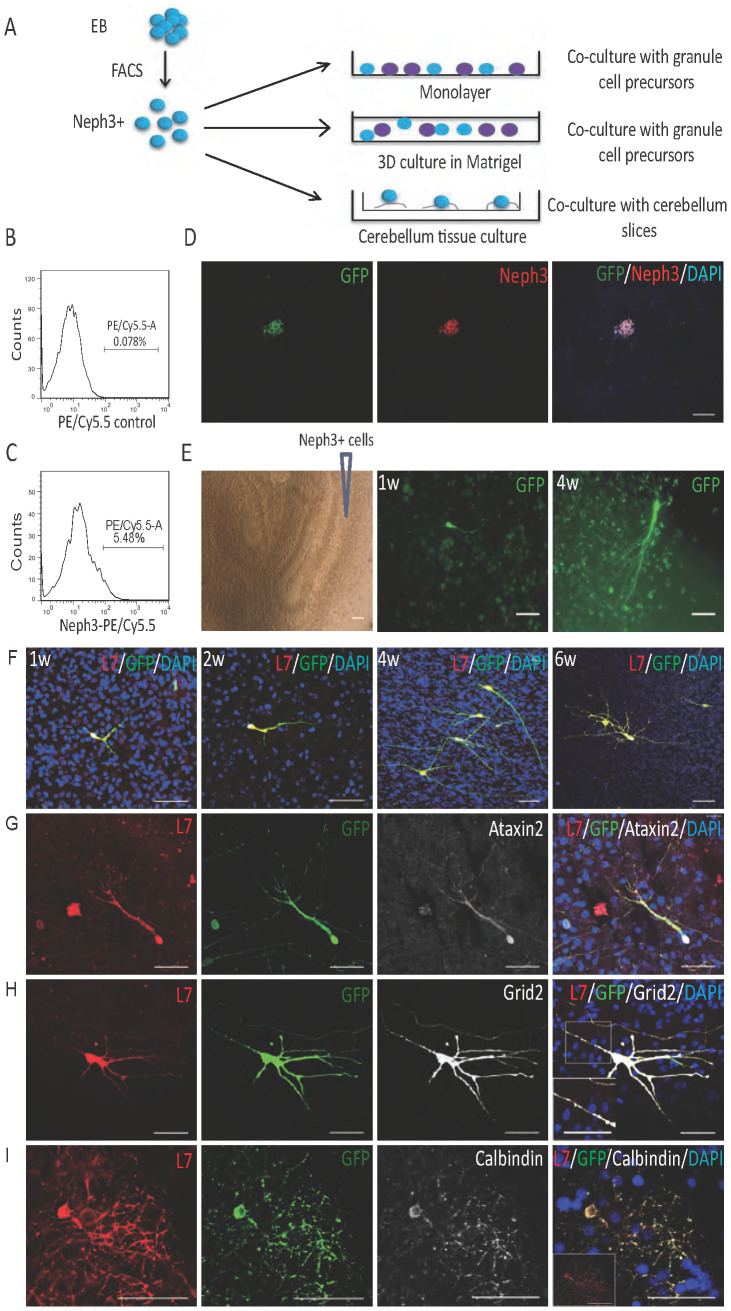
Neph3-positive cells co-cultured with rat cerebellar slices differentiate to cells with Purkinje cell morphology and marker expression. (A) Schematic representation of sorting and co-culture procedures. (B, C) FACS analysis of neural rosette cells on Day 20 of differentiation. (D) Immunofluorescence staining to confirm that the sorted cells were Neph3/GFP-double positive. Bar = 100 μm. (E) The sorted cells were co-cultured with cerebellum slices and examined at 1 week and 4 weeks. Bar = 100 μm. (F) Immunofluorescence staining of L7 and GFP on the sorted cells at different time points (1 week, 2 weeks, 4 weeks and 6 weeks in co-culture). Bar = 50 μm. (G–I) At 4 weeks in co-culture, L7+ cells also expressed other Purkinje cell markers Ataxin2 (G), Grid2 (H), and Calbindin (I). In Fig. 2H, a lower left inset was placed in the merged picture to amplify the marked region. A L7-positive cell with typical morphology was depicted as an inset in Fig. 2I. Bar = 50 μm. The immunostaining experiments were repeated at least three times.

Neph3 is a surface marker that can be used for sorting Purkinje progenitors. On Day 20, around 10% of cells stained positive for Neph3 by immunofluorescence. We used fluorescence activated cell sorting (FACS) to enrich for Purkinje progenitors. Culture at Day 20 were dissociated with either Accutase or Trypsin, followed by incubation with goat anti-Neph3 primary antibodies and PE-conjugated secondary antibodies (Biotin-conjugated anti-Neph3 and PE-conjugated anti-biotin antibodies were also tested). However, under none of the above conditions could we sort out any Neph3-postive cell population by using FACS. One possibility was that the enzymatic treatment may have cleaved off Neph3 molecules from cell surface. To test it, we incubated the culture with primary and PE-conjugated secondary antibodies and examined the staining under fluorescence microscope. The positive signals were indeed there. However, after enzymatic treatment with Accutase or Trypsin and two washes, all the positive signals were gone, confirming that Neph3 had shed off by the treatment. Therefore, we switched to non-enzymatic buffer for the dissociation step. Two different buffers were tested, non-enzymatic dissociation buffer from Sigma and cell dissociation buffer (enzyme free) from Life Technologies. At least 20 min treatment was required to obtain good single cell suspension. Both types of buffers were stressful to the cells and resulted in cell death. Relatively, cell dissociation buffer from Life Technologies was milder and was chosen for subsequent sorting experiments (Fig. 2A). Around 5% of total green fluorescence protein (GFP)-labeled cells were sorted as Neph3-positive population by using BD FACSAria-II cell sorter (Fig. 2B and C) and the collected cells were further confirmed to be Neph3-positive (Fig. 2D).

The sorted Neph3+ cells were cultured alone either on Millicell insert or Corning Synthemax-coated plates in Purkinje differentiation medium. When cultured on membrane inserts, less than 20% of the seeded cells survived after 7 days. The surviving cells displayed a round morphology without any processes (Fig. S2A). At 2 weeks, less than 1% cells survived which still exhibited a round morphology (Fig. S2B). Those few surviving cells did not go through further differentiation even after extended culture (>4 weeks). The sorted cells cultured on Synthemax-coated plates survived better (60–70% survival) but differentiated neither, similar to the cells cultured on the membrane inserts (data not shown).

Previous studies show that mouse Purkinje progenitors can further differentiate when co-cultured with isolated granule precursor cells^9^. Therefore, we isolated granule precursors from the rhombic lips of E15.5 SD rat embryos. Neph3+ cells were co-cultured with granule precursors as monolayer on poly-D-lysine-coated plates. However, the co-culture could not survive medium change and all the cells died after even change of 1/3 of total volume. We then tried to feed the cells with glucose every other day without medium change. In this way, the granule precursors cultured alone could readily survive for more than 4 weeks (Fig. S2C). In contrast, granule precursors and Neph3+ cells in co-culture resulted in change of medium color from red to yellow in 1 or 2 days, and most cells died within 1 week (Fig. S2D). Very few Neph3+ cells (GFP-positive) survived and showed Purkinje cell morphology with simple dendritic processes after 2 weeks of co-culture (Fig. S2E), while the granule precursors cultured alone could readily survive for more than 4 weeks.

We reasoned that culturing the Neph3+ and granule precursor cells in a 3-dimensional way in Matrigel may provide a more supportive niche, and therefore tested two culture methods. In the first one, the granule precursors were mixed with Matrigel, which was then cast into plate wells as a 3-dimensional structure. Sorted Neph3+ cells were seeded on top of the Matrigel containing granule precursors. In the second method, Neph3+ cells were mixed with granule precursors at different ratios (1:100, 1:200, 1:400, 1:1000, and 1:5000) in Matrigel before being cast into plate wells. However, under neither of the above conditions could the co-culture survive for more than 1 week, and no further differentiation of Neph3+ cells was observed (Fig S2 F–H).

During cerebellum development, the structural organization of different cell types, the electrical input from granule and/or other cells, as well as soluble factors produced by resident cells could be important to the differentiation and maturation of Purkinje progenitors. Simple co-culture of Purkinje progenitors with granule cells may have missed some key regulatory factors in such a process. Therefore, we tried culture of sorted Neph3+ cells on organotypic cerebellum slices (Fig. 2E). Due to the small size, slicing of prenatal mouse or rat cerebellum remains a technical challenge. We then used postnatal day 7–10 Spraque-Dawley (SD) or immunodeficient nude rat to generate cerebellar organotypic slices. Neph3+ cells were placed on the slice cultures at various densities from 200 to 10000 cells per slice, and cultured in differentiation medium for 1, 2, 3, 4, 5, or 6 weeks. Upon co-culture, the seeded Neph3+ cells started to change morphologies. By 1 week of co-culture, the cells had extended simple processes (Fig. 2F). By 4 and 6 weeks, the processes became longer and more complex (Fig. 2F). The seeded green cells at different time points stained positive for mature Purkinje cell markers L7, Ataxin 2 and Grid2 (Fig. 2G and H). Some of the cells showed mature Purkinje cell morphology with prototypical processes (Fig. 2I). No obvious difference was detected in differentiation of Purkinje cells on SD versus nude rat slices (data not shown).

### Human fetal cerebellum slices provide sufficient niche to achieve physiologically functional Purkinje neurons

Co-culture with rat cerebellum slices proved to be an efficacious means to generate Purkinje cells with the right morphology and marker expression pattern. Next we tested whether these cells possess electrophysiological functions. Seeded cells cultured on rat slices were subjected to patch clamping at various time points (1, 2, 3, and 4 weeks in co-culture). The resting membrane potentials varied in the range of −31 mV to −44 mV during 1–4 weeks (Fig. S3A), and the sodium currents increased over time at a holding potential of 0 mV (Fig. S3B). Nevertheless, no spontaneous post-synaptic current (PSC) or action potential was detected at all the time points examined. Even after injection of stepped currents, no action potential was observed (data not shown), suggesting that co-culture with rat cerebellar slices is not adequate to provide all the components for the assembly of electrophysiological machineries.

**Figure 3:**
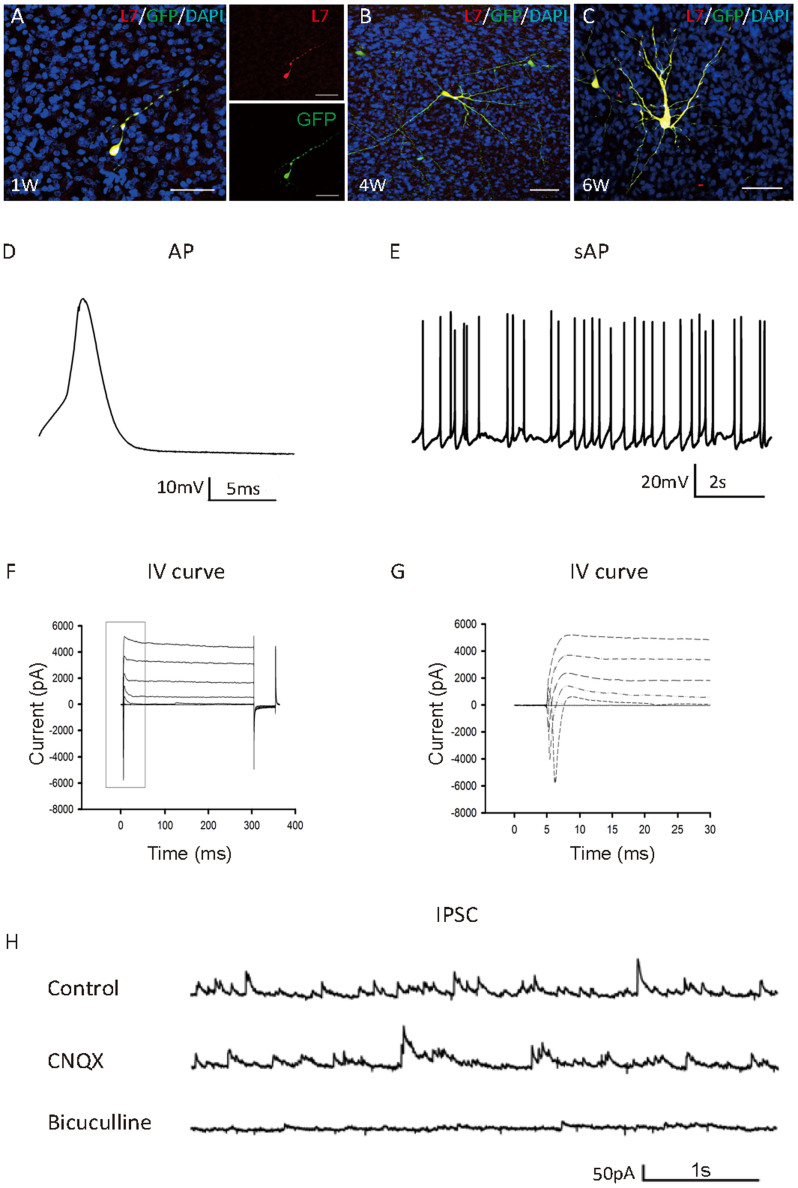
Purkinje progenitors co-cultured with human cerebellar slices mature to physiologically functional neurons. (A–C) Immunofluorescence staining of the sorted cells in co-culture with human cerebellar slices at different time points. Bar = 50 μm. (D) Single action potential was evoked by injecting short (4–9 ms) depolarizing current pulse (n = 22). (E) Spontaneous synaptic potentials at a holding current of 0 pA (n = 21). (F, G) Voltage-clamp recordings of the differentiated cells. Currents were elicited by stepping the potential from −60 to +60 mV at 20 mV intervals. (H) Spontaneous synaptic currents without (top), with CNQX (middle), and with CNQX + bicuculline (bottom) treatment (n = 7).

The failure to obtain physiologically functional Purkinje neurons by co-culture on rat slices may reflect a species difference between rodent and human cells. Differences in the gestation periods, time length required for cell maturation, and molecular cues for migration and maturation, etc. may be possible reasons accounting for morphologically normal Purkinje cells yet with deficient electrophysiological characteristics. If this is true, using human cerebellum slices may improve outcome. Fetal cerebellum slices (16–23 weeks of gestation) were established to address this issue. GFP-labeled Neph3+ human cells were seeded on the slices and co-cultured for 1 to 6 weeks (Fig. 3A–C). The resting membrane potentials varied in the range of −36.6 mV to −58.7 mV during 2–4 weeks (Fig. S3C), and the sodium currents also increased over time at a holding potential of 0 mV (Fig. S3D). The seeded cells started to show action potentials (8 out of 10 recorded cells) (Fig. 3D) at 2 weeks in co-culture. By 4 weeks in co-culture, 90% (9 out of 10) showed action potentials and 40% (4 out of 10) showed frequent spontaneous action potentials (sAP, Fig. 3D and E). Fig. 3F and G showed the currents over time at different holding potentials and Fig. 3H represented the inhibitory post-synaptic current (IPSC) trace of a recorded cell. The recorded cells were labeled with neurobiotin through the electrode, and later fixed and confirmed to be Purkinje cells (L7-positive, data not shown).

### Neph3-positive cells differentiate after engraftment into immunodeficient mice

**Figure 4:**
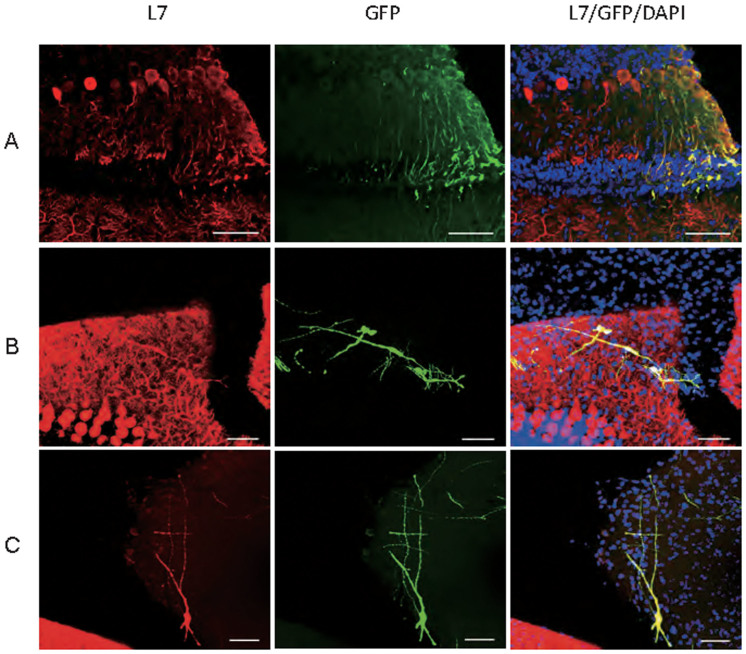
Neph3-positive cells differentiate and integrate *in vivo*. The sorted GFP-labeled Neph3+ cells were injected into the cerebella of newborn SCID mice. Four weeks later, the mice were sacrificed and GFP/L7-doulbe positive cells were found at the Purkinje layer (A), molecular cell layer (B) and non-cerebellum regions (C). Bar = 50 μm.

Obtaining Purkinje progenitors that are capable of differentiation and integration *in vivo* would be of clinical relevance. GFP-labeled sorted Neph3+ cells (2 × 10^4^ cells) were injected into the cerebellum of postnatal day 0 immunodeficient SCID mouse pups (n = 12). Four weeks later, the mice were sacrificed for analysis. GFP+/L7+ cells were found in two of the 12 SCID mice. About 90–100 GFP+/L7+ cells in each of the two mice were detected at different locations (Fig. 4A–C). Engrafted cells detected at the Purkinje layer showed cell bodies aligned with the endogenous Purkinje neurons, with complex dendrites extending to the molecular layer (Fig. 4A). Some transplanted cells ended in the molecular layer, displaying less complex dendrites oriented mostly parallel to the Purkinje cell layer (Fig. 4B). In some mice with missed targeting of injection, we still observed L7+ cells outside of cerebellum, for example, midbrain (Fig. 4C). The ectopic detection of L7+ engrafted cells suggested that the sorted Neph3+ cells had activated an intrinsic program of lineage specification.

## Discussion

In this study, we managed to differentiate human iPSCs to Purkinje progenitors by activating an endogenous pathway of EBs to produce morphogens that play central roles in cerebellum patterning. The Purkinje progenitors further matured to electrophysiologically functional Purkinje neurons by co-culturing with human fetal cerebellar slices. In addition, the progenitors could survive transplantation into immunodeficient mouse brains and differentiate to L7-positive Purkinje cells.

Purkinje cells are injured in genetic neurodegenerative diseases such as certain spinocerebellar ataxias (SCAs), and other medical indications such as toxic exposure and autoimmune diseases. The advent of iPSC technology has made it possible to directly study patient-specific neurons in a culture dish; and this has greatly advanced the research on disease etiology and mechanisms. However, the failure to obtain human iPSC-derived Purkinje cells has remained an obstacle to study cerebellum neurodegenerative diseases, for example, SCA2, in which Purkinje cells are the primary target of pathology. To our knowledge, this is the first time to differentiate human iPSCs to functional Purkinje neurons, and future studies will be conducted to use this method to model SCAs.

The resting membrane potentials of Purkinje cells co-cultured on SD rat cerebellar slices were −39.8 ± 10.6 mV, while those on human fetal slices were −58.7 ± 2 mV at 4 weeks (Fig. S3A and C); cells cultured on human cerebellar slices could fire spontaneous action potentials whereas cells on rat slices could not (Fig. 3D and E); synaptic connections were detected on seeded cells co-cultured with human brain slices but not those with rat ones (Fig. S3E). All these suggested that Neph3-positive progenitors seeded on human brain slices had proceeded to a more mature phenotype. It is still unclear what made it a permissive niche in human fetal cerebellar slices for the further maturation of Purkinje progenitors. One possibility was species difference. Human Purkinje cells naturally develop in a human cell environment and may prefer to mature on a human slice versus on a rodent one. Another possibility was the difference in slice ages. Due to the technical challenges in preparing fetal cerebellar slices (too small in size), we could only use slices prepared from postnatal rats, which was late in developmental stage compared to the human cerebellar slices used. Future studies are required to find the optimal time window of cerebellar slices that can provide the best environment for Purkinje cell maturation.

In summary, the present study, for the first time to our knowledge, reported the differentiation of human iPSCs into Purkinje neurons *in vitro* and *in vivo*, which may break the bottleneck in the research field and make it possible to study patient-specific Purkinje neurons. This work will facilitate disease modeling of genetic SCAs in a culture dish and regenerative studies targeting cerebellar diseases with Purkinje cell injury as major or part of pathology.

## Methods

### Ethics statement

The fibroblast cells used in iPSCs induction were obtained from a Chinese family according to standard protocol. Human fetal cerebellum slices were prepared from the cerebellum of electively aborted embryos (16–23 weeks post conception). Informed consent has been obtained from all subjects. All protocols used in this study were approved by Ethics Committee of Xuanwu Hospital, Capital Medical University, and the methods were carried out in accordance with the approved guidelines.

### Isolation of fibroblasts and generation of iPSCs

Fibroblasts were obtained from individuals of a Chinese family with written consent. Fibroblasts were prepared according to the method published previously^14^ and iPSCs were generated using a protocol developed by Yamanaka and colleagues^15^. Human ES-like colonies appeared after 14–21 days post infection. Colonies were manually picked and transferred onto Matrigel (BD Bioscience, New Jersey, USA)-coated 24-well plates and cultured in mTesR medium (Stemcell Technologies, Vancouver, Canada) for expansion. For passaging, iPSCs were incubated with Accutase (GE Healthcare Life Sciences, Fairfield, USA) at 37°C for 2 min, washed with PBS, and scraped off from the plate by using the tip of a 2 ml pipette. Then the cells were collected into centrifuge tube, spun down, and re-suspended with fresh medium, and split in a ratio of 1:6.

### Differentiation of iPSCs to Purkinje progenitors

The procedure was modified from a published mouse study^9^. Prior to differentiation, iPSCs were infected with lentiviral vectors expressing GFP (FUGW, Addgene Cat.^#^: 14883). iPSCs stably expressing GFP were used in the following differentiation procedures. On day 0, iPSCs were treated with Collagenase IV (Sigma-Aldrich, St. Louis, Mo, USA) at 37°C for 15-20 min. Then the cells were washed with PBS, and scraped with the tip of 2 ml pipette into small clusters, which would re-aggregate into embryonic bodies (EBs) in gfCDM medium plus 7 μg/ml insulin after overnight incubation. The gfCDM medium consists of Isocover's modified Dulbecco's medium/Ham's F-12 (Life Technologies, California, USA) 1:1, chemically defined lipid concentrate (Life Technologies), penicillin/streptomycin, monothioglycerol (450 mM, Sigma-Aldrich), apo-transferrin (15 μg/ml, Merk Millipore, Billerica, USA) and BSA (5 mg/ml, GE). From day 1 onward until day 20, 20 ng/ml Fgf2 was added to gfCDM medium plus insulin. From day 7 to 10, 10 μg/ml cyclopamine (Sigma-Aldrich) was added to induce dorsalization. On day 10, gfCDM EBs were transferred to poly-D-lysin (50 μg/ml, Sigma-Aldrich)/laminin (5 μg/ml, Roche, Basel, Switzerland)-coated 6-well plates to allow attachment and formation of rosette-like structures.

### AP staining and immunocytochemistry

AP staining was performed using SIGMAFAST BCIP/NBT staining kit (Sigma-Aldrich). Immunocytochemical staining was performed according to the method published before^9^. Primary antibodies and working dilutions were listed in Table S1. The staining results were repeated at least three times. The representative pictures were showed in related figures.

### Cell dissociation and sorting

Culture at Day 20 were dissociated with cell dissociation buffer (enzyme free, Life Technologies, Catalog No. C5789) for 20 min to obtain good single cell suspension. The dissociated cells were filtered through a cell strainer (40 μm, BD bioscience) and labeled with anti-Neph3 monoclonal antibody (R&D) which had been pre-conjugated with secondary antibodies by using Lightning-link PE/Cy5.5 DIY antibody labeling kit (Innova Bioscience, Cambridge, UK). Neph3-positive cells were sorted by using FACSAria II (BD bioscience) and the data analyzed using the FACSDiva software. A sample of sorted Neph3+ cells was re-analyzed by FACS and immunocytochemistry. In an attempt to achieve further differentiation, the sorted cells were cultured under the following different conditions. 1) To plate Neph3+ cells on the membrane of Millicell cell culture insert (Merk Millipore); 2) To plate the sorted cells on Corning Synthematrix plates; 3) Co-culture with cerebellum granule cells derived from the upper rhombic lips of E15.5 SD rats; 4) Co-culture with cerebellum slices derived from SD rats; 5) Co-culture with cerebellum slices derived from human fetus. In addition, Neph3+ cells were transplanted into the cerebellar region of newborn immunodeficient mice in *in vivo* experiments.

### Organotypic cerebellum slice culture

Animal experiments were performed in accordance with the Institutional Animal Care and Use Committee guidelines at the Capital Medical University. Human fetal cerebellum slices were prepared from the cerebellum of electively aborted embryos (16-23 weeks post conception). The protocol used in this study was modified from a previous report^16^. Briefly, postnatal day 10 (P10) SD or nude rat pups were deeply anesthetized when the brains were rapidly removed. The cerebellum was dissected out and serial sagittal slices (350 μm) were prepared using a McIlwain tissue chopper (The Mickle Laboratory Engineering Co. Ltd, Gomshall, Surrey, UK), and transferred to cold sterile Hank's balance salt solution with D-glucose (5 mg/mL). The slices were separated under a dissecting microscope, and carefully transferred onto a Millicell membrane insert (0.4 μm, Merk Millipore) set in a 6-well plate. From Day 1 to 7, slices were maintained in 1 ml of slice medium consisting of 50% Neurobasal medium (Life Technologies), 25% horse serum, and 25% Hanks' balanced salt solution supplemented with 5 mg/ml D-glucose and 2 mM L-glutamine at 37°C, in 5% CO_2_ incubators. Medium was changed every other day. On day 7, Neph3+ cells labeled with GFP were added onto the surface of slices at various densities, and the slice medium was changed to Purkinje cell medium (DMEM/F12 + 10% FBS + 1% N2 (Life Technologies) + 7 g/l D-Glucose + 30 nM L-3,3′,5-Triiodothyronine^17^ (Merk Millipore) + 10 ng/ml IGF-1^18^ (Peprotech, Rocky Hill, USA) + Estrogen^19^ (Sigma-Aldrich) + Penicillin/Streptomycin.

### PCR

Total RNA was purified using RNeasy Mini Kit (Qiagen, Duesseldorf, Germany) according to the manufacturer's instructions. For genomic DNA PCR, genomic DNA was purified with the Wizard Plus Minipreps DNA Purification System (Promega, Wisconsin-Madison, USA). PCR was performed using rTaq DNA polymerase (Takara, Tokyo, Japan) and the PCR primers were listed in Table S2. The experiment was repeated at least 3 times.

Quantitative PCR was performed with GoTaq qPCR master mix system (Promega) and analyzed in the LightCycler 480 system (Roche, Basel, Switzerland). The primers were also listed in Table S2. Data were normalized to 18sRNA and hypoxanthine phosphoribosyltransferase (HPRT) expressions. The values shown in the graph represent mean ± S.E.M. Every experiment was repeated at least 3 times.

### Bisulfate sequencing

Genomic DNA was treated with CpGenome DNA modification kit (Merk Millipore) according to the manufacturer's introductions. Treated DNA was purified with DNA purification kit (Qiagen). The promoter regions of human OCT4 and NANOG were amplified by PCR. Then the PCR products were subcloned into PMD20-T vectors (Takara). Ten clones of each sample were sequenced. Primers were listed in Table S2.

### Teratoma formation

iPSCs were treated with Collagenase IV and harvested using cell scrapers. Cell pellets were re-suspended in Matrigel and transferred to animal room on ice. Cells from 6 wells of 6-well plate were injected into the hind limb muscle of a SCID/Beige mouse. After 6-8 weeks, tumors were dissected and fixed with 4% paraformaldehyde.

### Cell transplantation

Neph3+ cells sorted on Day 20 were re-suspended in HBSS with 5 mg/ml D-Glucose and 20 ng/ml Fgf2. The newborn SCID/Beige mice were placed on ice for anaesthetization. One μl of cell suspension (2 × 10^4^ cells) was injected into the cerebellum region using a glass micropipette. Four weeks after injection, the mice were sacrificed and the cerebellum was dissected and sectioned at 40 μm thickness.

### Electrophysiology analysis

Whole-cell patch-clamp recordings were performed on GFP-positive Purkinje cell-like cells co-cultured with cerebellum slices. The extracellular solution included (in mM): 126 NaCl, 2.5 KCl, 1.25 NaH2PO4, 1.8 CaCl2, 1.2 MgSO4, 25 NaHCO3, 15 D-glucose. The pH was maintained at 7.3 by bubbling with 95% O2:5% CO2. The patch pipette solution contained the following (in mM): 140 K-gluconate, 5 KCl, 0.2 ethyleneglycol-bis-(b-aminoethyl ether)-N,N,N%,N%,-tetraacetic acid (EGTA), 2 MgCl2 and 10 HEPES. The pH was adjusted to 7.2 with 1 M KOH. All experiments were done in a closed-loop, heated perfusion chamber (~31°C). Drugs were applied to the ACSF reservoir and allowed to perfuse onto the slice using a microperfusion device (MPS-2; INBIO, Wuhan, China) with a fast exchange time (<100 ms) among eight channels. Based on the steady flow rate, we reasoned that 2 min were sufficient to equilibrate the recording chamber with drugs at their appropriate concentrations.

Electrode resistance ranged from 5 to 7 MΩ. Tight seals (~GΩ) were obtained on cell bodies before rupturing the membrane with negative pressure. Single action potential was evoked by injecting short (4–9 ms) depolarizing current pulses (in current clamp). To detect GABA receptor-mediated inhibitory post-synaptic currents (IPSCs), the cells were monitored at a holding potential of 0 mV (close to the reversal potential for glutamate-activated currents). Membrane potential values were corrected for the liquid junction potential (~10 mV). In experiments, 20 μM bicuculline (Sigma-Aldrich) was used to block inhibition.

The whole cell recording was conducted with HEKA EPC-10 patch-clamp amplifier with associated software (PatchMaster, HEKA Electronic Inc., Germany). The currents were typically digitized at 10 KHz. Macroscopic records were filtered at 2.9 KHz. Data were analyzed with Clampfit software (version 10.0; Axon Instruments) and Sigmaplot (Systat Software, Inc., Chicago, IL) software. The representative traces were showed in Fig. 3D–H. The statistical data about the resting potentials and Na+ currents were presented as mean ± S.E.M in Fig. S3. For data obtained from the same cell, paired t-test was used whereas unpaired t-tested was adopted for analyzing data collected from a group of cells.

## Author Contributions

Z.C. designed the study, analyzed data, provided financial support and wrote the main manuscript text. S.W. analyzed data and prepared Figures 1–4, and Figures S1–3. N.P. prepared the electrophysiology data of Figure 3. B.W., L.F., G.S., J.A. and Z.L. conducted the experiments and collected the data. C.W., W.Z., Y.G. and P.C. provided the patient materials. Z.Q.X. provided administrative support. Y.A.Z. provided financial support. All authors reviewed the manuscript.

## Supplementary Material

Supplementary Information Supplementary information

Supplementary Information Supplementary Figure 1

Supplementary Information Supplementary Figure 2

Supplementary Information Supplementary Figure 3
