# Supplementary material for: Differentiation of human induced pluripotent stem cells to mature functional Purkinje neurons
Source: Sci Rep. 2015 Mar 18;5:9232. doi: 10.1038/srep09232 (PMC4363833; doi:10.1038/srep09232)
Supplement: Supplementary Information [file srep09232-s1.pdf]

Differentiation of human induced pluripotent stem cells to mature functional Purkinje neurons

Shuyan Wang[1,2,3], Bin Wang[1], Na Pan[5], Linlin Fu[1], Chaodong Wang[6], Gongru Song[1], Jing An[1], Zhongfeng Liu[1], Wanwan Zhu[1], Yunqian Guan[1], Zhi-Qing David Xu[5], Piu Chan[3,6] , Zhiguo Chen[1,2,3,*], Y. Alex Zhang[1,4]

[1] Sanofi-Xuanwu Joint Lab for Regenerative Medicine, Xuanwu Hospital, Capital Medical University, Beijing, 100053, China

[2] Center of Neural Injury and Repair, [3] Center of Parkinson's Disease, Beijing Institute for Brain Disorders, Beijing, China

[4] China R&D Center, Sanofi, Beijing 100022, China

[5] Department of Neurobiology, Beijing Key Laboratory of Major Brain Disorders, Beijing Institute of Brain Disorders, Capital Medical University, Beijing, 100069, China

[6] Department of Neurobiology, Xuanwu Hosptial, Capital Medical University, Beijing, 100053, China

***Correspondence and requests for materials should be addressed to Zhiguo Chen**

Zhiguo Chen, Ph.D.

Email: chenzhiguo@gmail.com

Supplemental Information

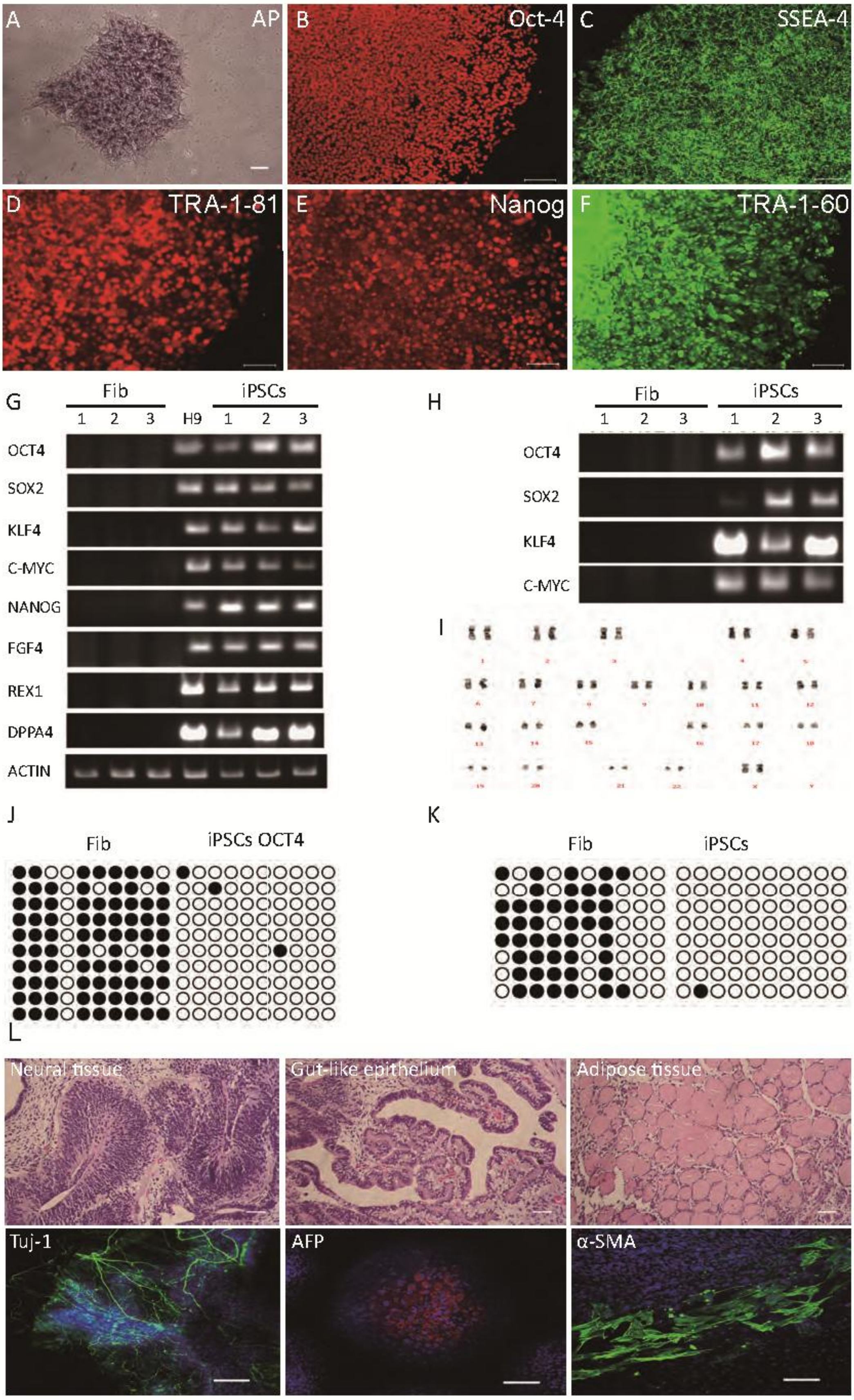

Figure S1. Generation and characterization of iPSCs. Related to Figure 1.

(A-F) Immunofluorescence staining for AP (A), Oct4 (b), SSEA-4 (C), TRA-1-81 (D), Nanog (E) and TRA-1-60 (F). Bar=100 μm.

(G) Expression of pluripotent genes detected by RT-PCR. Primers for OCT4, SOX2, KLF4 and c-MYC were specific for endogenous genes.

(H) PCR of genomic DNA confirmed that the transgenes had incorporated into the genome.

(I) The iPSC clones showed normal karyotype.

(J, K) Bisulfate sequencing of promoter regions for endogenous OCT4 and NANOG genes. Open and closed circles indicated unmethylated and methylated CpG islands, respectively.

(L) Hematoxylin and Eosin staining of terotoma following injection of iPSCs into immunodeficient mice (upper pannel) and *in vitro* differentiation of iPSCs to 3-germ layer cells (lower panel). Bar=100 μm.

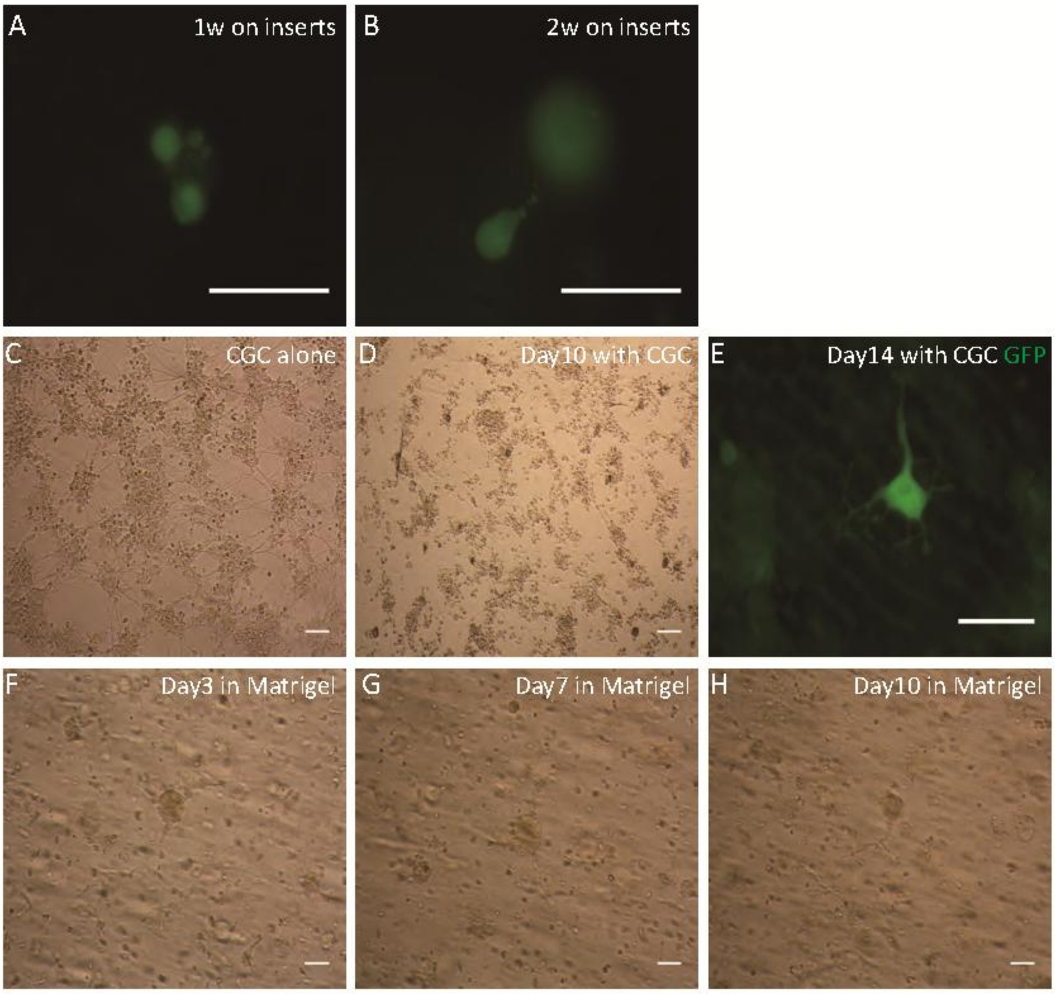

Figure S2. Neph3+ cells fail to survive or differentiate when cultured alone or with cerebellar granule cells. Related to Figure 2.

(A, B) Sorted neph3+ cells cultured on a whole membrane insert. On day 7 of differentiation, more than 80% of cells died and the surviving cells showed a round morphology (A). On day 14, less than 1% of cells survived and displayed an undifferentiated morphology (B). Bar=50 μm.

(C) Cerebellar granule cells could survive for more than 4 weeks when cultured alone. Bar=100 μm.

(D) In co-culture with Neph3+ cells, most granule cells died within 1 week. Bar=100

μm.

(E) After most Granule cells died, very few neph3+ cells (GFP-positive) survived and showed Purkinje cell morphology with very simple dendritic tree. Bar=50 μm

(F-H) Sorted Neph3+ cells failed to differentiate when co-cultured with cerebellar granule cells in a 3-dimentional manner. Bar=100 μm.

CGC: cerebellar granule cells.

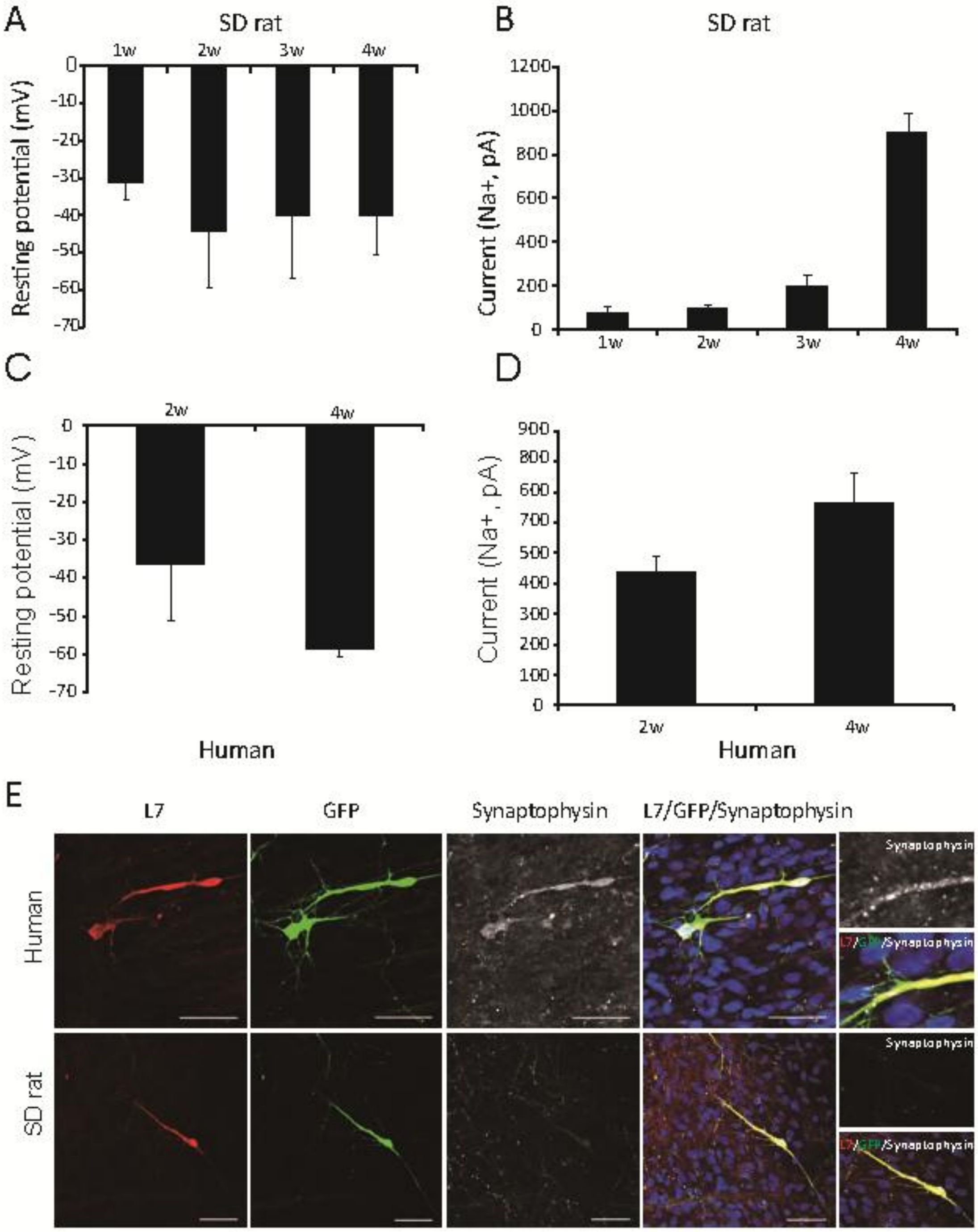

Figure S3. Electrophysiological characteristics of Purkinje cells co-cultured with SD rat and human fetal cerebellum slices. Related to Figure 3.

(A) Resting membrane potentials of recorded Purkinje cells on SD rat cerebellum slices at different time points.

(B) Sodium currents increased over the course of differentiation on SD rat cerebellum

slices.

(C) Resting membrane potentials of recorded Purkinje cells on human fetal cerebellum slice at different time points.

(D) Sodium currents increased over the course of differentiation on human fetal cerebellum slices.

(E) Immunocytochemical staining of Synaptophysin and L7 for co-culture on human fetal cerebellum slices and on SD rat cerebellum slices. Bar=50 μm.

Table S1. Antibodies used for immunohistochemistry analyses. Related to Figures 1 and 2.

Antigen	Company	Catalog Number	Working Dilution
Oct3/4	Millipore	MAB4401	1:200
SSEA-4	eBioscience	14-8843-80	1:200
Nanog	eBioscience	14-5769-80	1:200
TRA-1-60	eBioscience	12-8863-80	1:200
TRA-1-81	eBioscience	14-8883-80	1:200
α-SMA	Abcam	ab8207	1:100
AFP	eBioscience	14-6583-80	1:200
E-Cadherin	eBioscience	53-3249	1:400
Kirrel2 (Neph3)	R&D	BAF2564	1:400
En2	Abcam	ab28731	1:400
Ptf1a	R&D	AF6119	1:400
Ki67	Novocastra	NCL-L-Ki67-MM1	1:500
FUSSEL18 (Corl2)	Abgent	AP11997b	1:50
L7 (PCP-2)	Santa Cruz	sc-49072	1:200
L7 (PCP-2)	Santa Cruz	sc-137064	1:200
GFP	Santa Cruz	sc-9996	1:200
GFP	Life Technologies	A11122	1:500
Ataxin2	Santa Cruz	sc-18477	1:100
Grid2	Abcam	Ab101886	1:400
Synaptophysin	Sigma	S5768	1:200
Tuj-1	Millipore	MAB1637	1:400
Calbindin	CST	2173S	1:200

Table S2. Sequences of primers for PCR, RT-PCR and Real-time PCR. Related to Figures 1 and 2.

**Primer Names**	**Primer sequences (5’-3’)**
**RT-PCR primers used for characterization of iPSCs**	
OCT4 endo F	GACAGGGGGAGGGGAGGAGCTAGG
OCt4 endo R	CTTCCCTCCAACCAGTTGCCCCAAAC
SOX2 endo F	GGGAAATGGGAGGGGTGCAAA AGAGG
SOX2 endo R	TTGCGTGAGTGTGGATGGGAT TGGTG
KLF4 endo F	ACGATCGTGGCCCCGGAAAAGGACC
KLF4 endo R	TGATTGTAGTGCTTTCTGGCTGGGCTCC
c-MYC endo F	GCGTCCTGGGAAGGGAGATCCGGAGC
c-MYC endo R	TTGAGGGGCATCGTCGCGGGAGGCTG
NANOG F	CAGCCCCGATTCTTCCACCAGTCCC
NANOG R	CGGAAGATTCCCAGTCGGGTTCACC
GDF3 F	CTTATGCTACGTAAAGGAGCTGGG
GDF3 R	GTGCCAACCCAGGTCCCGGAAGTT
REX1 F	CAGATCCTAAACAGCTCGCAGAAT
REX1 R	GCG TACGCAAAT TAA AGT CCAGA
FGF4 F	CTACAACGCCTACGAGTCCTACA
FGF4 R	GTTGCACCAGAAAAG TCAGAG TTG
ESG1 F	ATATCCCGCCGTGGG TGAAAG TTC
ESG1 R	ACTCAGCCATGGACTGGAGCATCC
DPPA4 F	GGAGCCGCCTGCCCT GGAAAATTC
DPPA4 R	TTTTTCCTGATATTCTATTCCCAT
DPPA2 F	CCGTCCCCGCAATCT CCTTCCATC
DPPA2 R	ATGATGCCAACATGGCTCCCGGTG
**Bisulfate sequencing primers**	
meth *OCT-4* F	GAGGTTGGAGTAGAAGGATTGTTTTGGTTT
meth *OCT-4* R	CCCCCCTAACCCATCACCTCCACCACCTAA
meth *NANOG* F	TGGTTAGGTTGGTTTTAAATTTTTG
meth *NANOG* R	AACCCACCCTTATAAATTCTCAATTA
**Primers for genomic DNA PCR**	
OCT4 Tg genomic F	CCCCAGGGCCCCATT TTGGTACC
SOX2 Tg genomic F	GGCACCCCTGGCATGGCTCTTGGCTC
c-MYC Tg genomic F	CAACAACCGAAAATGCACCAGCCCCAG
pMXs R	TTATCGTCGACCACT GTGCTGCTG
PMXs L-3205	CCCTTTTTCTGGAGACTAAATAAA
**RT-PCR primers used in Purkinje precursor differentiation**	
EN2 F	CGGCTCCAAGACGCTCTCGC
EN2 R	CTGGGACCTGAAGAAGGCCGGT
FGF8 F	CCTACCAACTCTACAGCCGC
FGF8 R	ACTCGGACTCTGCTTCCAAA

WNT1 F	GGTTTCTGCTACGCTGCTG
WNT1 R	TAAGCAGGTTCGTGGAGGAG
**Primers used for quantitative PCR**	
18s RNA F	ACTCAACACGGGAAACCTCA
18S RNA R	AACCAGACAAATCGCTCCAC
HPRT F	GCTATAAATTCTTTGCTGACCTGCTG
HPRT R	AATTACTTTTATGTCCCCTGTTGACTGG
EN2 F	TACTCGGACCGGCCTTCTTC
EN2 R	AACTCGGCCTTGAGCCTCTG
WNT1 F	GGAACTGTCCCACTGCTCCA
WNT1 R	GCGGAGGTGATAGCGAAGATAAAC
LHX1 F	GCGTCCAGTGCTGTGAATGTAA
LHX1 R	GAAGCAGTTCAGGTGAAACACTTTG
OTX1 F	TCGGCTTGGCCTACACATTCTATAC
OTX1 R	GGGCTCAAACAGCGGATCA
FGF8 F	GGCCTCTACATCTGCATGAACAA
FGF8 R	GGGTGAAGGCCATGTACCA
NEPH3 F	CGAGGTCCTGAAGAAGAGGAGAC
NEPH3 R	ACACTGACTCCTCGGACCTTGT
LHX5 F	ACGAGAACAAGTTCGTGTGCAAAG
LHX5 R	AACTGCGGTCCGTACAGGATG
