# Supplementary figures and images for: Differentiation of human induced pluripotent stem cells to mature functional Purkinje neurons
Source: Sci Rep. 2015 Mar 18;5:9232. doi: 10.1038/srep09232 (PMC4363833; doi:10.1038/srep09232)

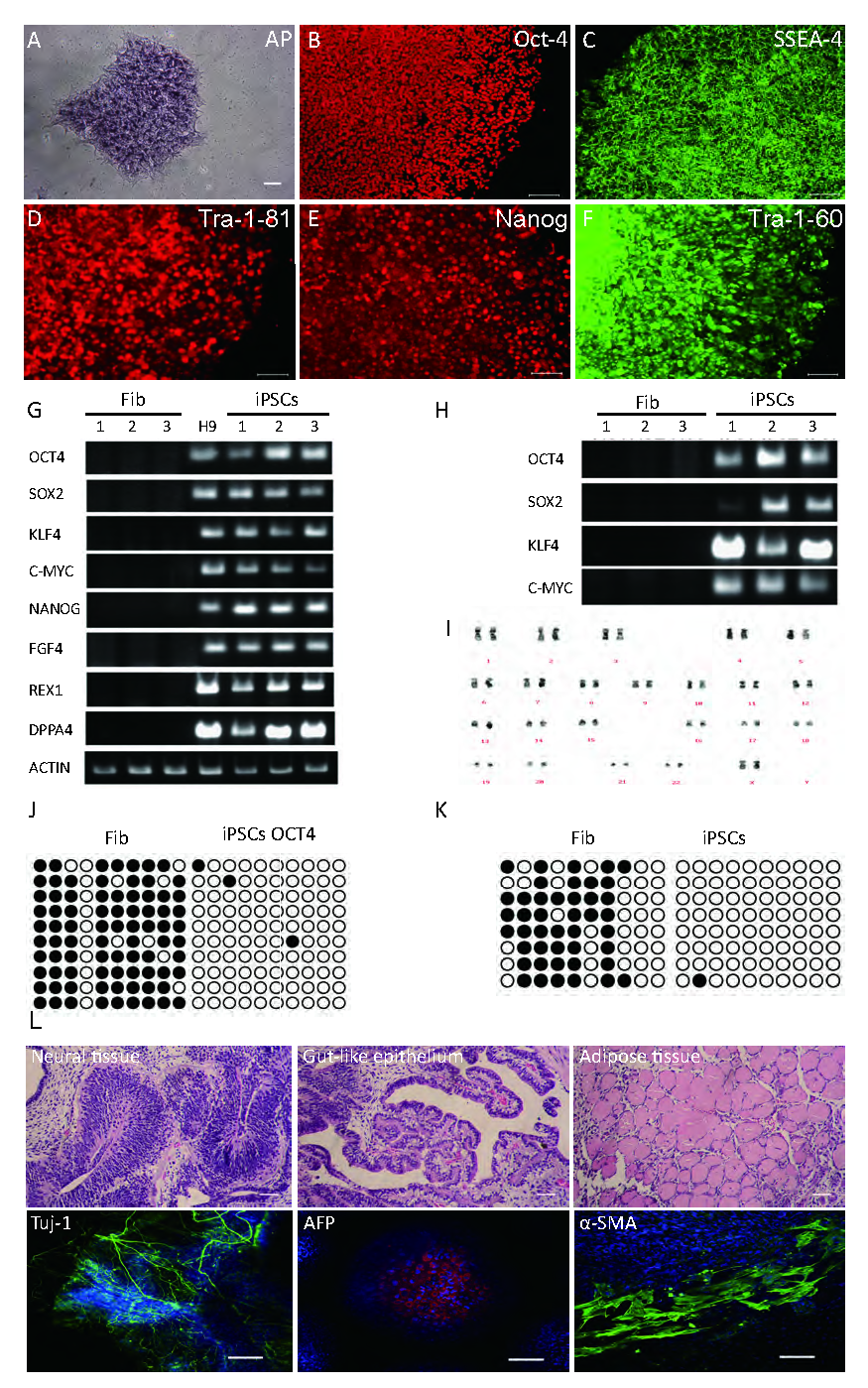

Supplement: Supplementary Information — Supplementary Figure 1 [file srep09232-s2.tiff]

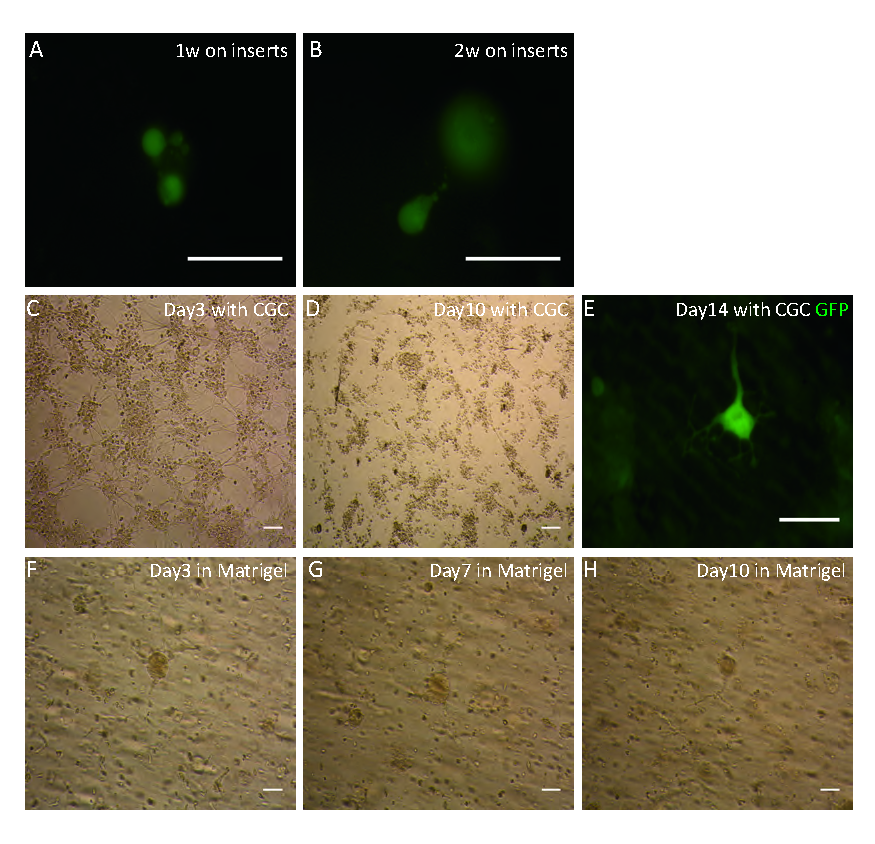

Supplement: Supplementary Information — Supplementary Figure 2 [file srep09232-s3.tiff]

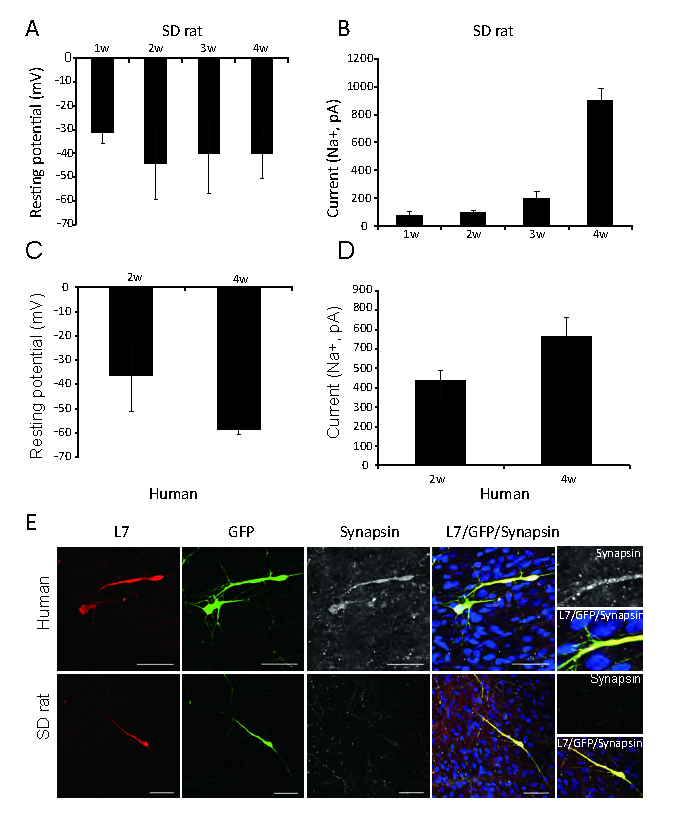

Supplement: Supplementary Information — Supplementary Figure 3 [file srep09232-s4.tiff]
